# Characteristics of Particles Emitted from Waste Fires—A Construction Materials Case Study

**DOI:** 10.3390/ma15010152

**Published:** 2021-12-26

**Authors:** Jan Stefan Bihałowicz, Wioletta Rogula-Kozłowska, Adam Krasuski, Małgorzata Majder-Łopatka, Agata Walczak, Mateusz Fliszkiewicz, Patrycja Rogula-Kopiec, Tomasz Mach

**Affiliations:** 1 Institute of Safety Engineering, The Main School of Fire Service, 52/54 Słowackiego Street, 01-629 Warsaw, Poland; wrogula@sgsp.edu.pl (W.R.-K.); akrasuski@sgsp.edu.pl (A.K.); mmajder@sgsp.edu.pl (M.M.-Ł.); 2 Faculty of Safety Engineering and Civil Protection, The Main School of Fire Service, 52/54 Słowackiego Street, 01-629 Warsaw, Poland; awalczak@sgsp.edu.pl (A.W.); mfliszkiewicz@sgsp.edu.pl (M.F.); 3 Institute of Environmental Engineering, Polish Academy of Sciences, 34 M. Skłodowska-Curie St., 41-819 Zabrze, Poland; patrycja.rogula-kopiec@ipis.zabrze.pl; 4 Faculty of Environmental Engineering, Wroclaw University of Science and Technology, Plac Grunwaldzki 13, 50-377 Wrocław, Poland; tomasz.mach@pwr.edu.pl

**Keywords:** volume size distribution, mass size distribution, density, pinewood, laminated particle board, polyurethane, poly(methyl methacrylate)

## Abstract

This study aimed to determine the relative densities of populations of particles emitted in fire experiments of selected materials through direct measurement and parametrization of size distribution as number (NSD), volume (VSD), and mass (MSD). As objects of investigation, four typical materials used in construction and furniture were chosen: pinewood (PINE), laminated particle board (LPB), polyurethane (PUR), and poly(methyl methacrylate) (PMMA). The NSD and VSD were measured using an electric low-pressure impactor, while MSD was measured by weighing filters from the impactor using a microbalance. The parametrization of distributions was made assuming that each distribution can be expressed as the sum of an arbitrary number of log-normal distributions. In all materials, except PINE, the distributions of the particles emitted in fire experiments were the sum of two log-normal distributions; in PINE, the distribution was accounted for by only one log-normal distribution. The parametrization facilitated the determination of volume and mass abundances, and therefore, the relative density. The VSDs of particles generated in PINE, LPB, and PUR fires have similar location parameters, with a median volume diameter of 0.2–0.3 µm, whereas that of particles generated during PMMA burning is 0.7 µm. To validate the presented method, we burned samples made of the four materials in similar proportions and compared the measured VSD with the VSD predicted based on the weighted sum of VSD of raw materials. The measured VSD shifted toward smaller diameters than the predicted ones due to thermal decomposition at higher temperatures.

## 1. Introduction

Waste disposal is an emerging problem in developed [1] as well as developing [2] countries. Even though many efforts have been made to limit waste production, the effects remain limited [3] Although many types of waste can be recycled and successfully used in different applications [4], recycled materials are often more expensive and hence less attractive for the industry than raw materials [5,6]. However, one of the most popular methods of waste treatment is landfilling [7], which has a negative effect on the environment [8]. Incineration is another important method, but it has negative social effects because waste incineration induces a false social belief that any burning of waste material is allowed. Household waste, especially in non-urban areas, is burned in bonfires in the backyard [9,10,11,12] or in barrels [13], especially in North America. The burning of waste at landfills or in stoves is also of significance [14] because the ion composition of the particulates indicates that solid waste is used as a fuel. Furthermore, the open burning of materials containing chlorine generates dioxins during incomplete burning [15]. Small-scale open burning waste incinerators emit more than 200 times the amount of dioxins than municipal waste incinerators [16], due to uncontrolled burning and lack of air pollution control [17]. Moreover, the open burning of waste generates greenhouse gases (GHGs) [18], which are not considered in global GHG inventories [19]. Furthermore, the open burning of waste produces volatile and semi-volatile organic compounds (VOCs and SVOCs), polycyclic aromatic hydrocarbons (PAHs), and polychlorinated dibenzofurans (PCDD/Fs). The emission factors of more than 160 pollutants and toxic substances generated during open burning were previously determined [20,21,22,23,24,25].

One of the most common sources of open waste burning is the burning of crop residues. Many recent studies have focused mostly on the number size distributions (NSDs) of particles emitted during the burning of crops. Investigations have been performed on chili and perilla crop residues [26], rice straw [27], and crop straw [28]. Nevertheless, waste burning in rural areas is not limited to these materials [29]. There is a saying in Poland that “waste in Poland can be divided into non-flammable and flammable waste and flammable waste can be further divided into waste that can be burned during the day and that during the night” [30,31]. This attitude signifies that almost everything can be burned in stoves or bonfires in backyards. Although recycling centers were introduced in every municipality (Local Administrative Unit level 2 [32]), the limited hours of operation of these centers and distance from the settlements have led to the inadequate disposal of furniture and construction materials.

Wood is the most commonly used material for the construction of single-family houses and is the main substance utilized for furniture [33]. The most popular tree used in the East European Plain, North European Plain, and Baltic Shield belongs to the *Pinus* spp. [34,35]. The mechanical properties and the heat properties of pinewood (PINE) have been well investigated [36,37,38]. Therefore, PINE is commonly used for construction purposes. Because of their popularity in furniture construction, PINE and chemically treated PINE have been the focus of indoor air quality studies [39]. PINE is also used as firewood.

The gaseous products emitted by the thermal decomposition of PINE are well known [40]. However, the NSD and mass size distribution (MSD) of the particles generated during PINE burning/combustion are known only with respect to furnace burning [41]. There remains a need to characterize the particle size distribution in the case of partial thermal decomposition such as in open burning, bonfires, or poorly ventilated heating stoves. In addition to plain wood, furniture can also be made from structural panels, such as laminated particle boards (LPBs) [42]. Regarding LPBs, the combustion properties LPB [43], heat release rates [44], and the yield of fire products [45] are well understood; however, particle emission remains to be investigated.

Polyurethane (PUR) is commonly used for the upholstery padding of furniture and its heat release rates [44] and fire toxicity [46] are well known. The NSD of particles generated by the thermal degradation of polyurethane has been investigated with respect to indoor air quality [47], but not with respect to burning.

Poly (methyl methacrylate) (PMMA) is used in households for decoration purposes (as a safe glass replacement) in picture frames, art objects, and aquarium walls. The burning of PMMA has been the object of many studies [48,49,50,51] since its use for the construction of objects contributed to the Summerland disaster [52].

In the present work, the size distributions of the particles generated by the burning of selected materials related to building fires and open burning were analyzed. The results provide information on the physical characteristics of populations of particles such as volume size distribution (VSD) and MSD and the possibilities of determining the relative densities of particles generated during PINE, LPB, PUR, and PMMA fires. The characterization of the size distributions of particles facilitates the identification of pollution sources [53,54]. The investigated materials can be burned either intentionally in waste fires or accidentally during residential building fires. Therefore, the results of this study can be applied to identify the origin of particles from both waste and residential fires. Moreover, it is important to determine the size distribution of the particles from fires to assess the health impact related to the inhalation of those particles [55,56,57].

This work includes an original method for determining the relative densities of populations of particles generated during fire experiments. The NSD and VSD were determined using low-pressure electric impactor measurements, and the MSD was obtained using gravimetric measurements. The crucial point in determining the density is to fit the densities in such a way that the parametrized VSD multiplied by the density equals the MSD.

## 2. Materials and Methods

### 2.1. General Description

Four different materials, that is, PINE, LPB, PUR, and PMMA, and a mixture of all materials (MIX), were used in our experiments. The experiments were conducted in a closed 75 m^3^ chamber, which was designed to evaluate fire detection systems. The sample was ignited, burned for a given time, and extinguished. Subsequently, the chamber was ventilated. During this process, the particle number concentration was measured using an impactor, that is, Dekati^®^ High-Temperature ELPI+^®^ (HT-ELPI, Dekati, Kangasala, Finland). The components of the impactor and substrates were prepared for experiments in a laboratory dedicated to environmental monitoring measurements at the Institute of Safety Engineering. Substrates from the manufacturer of the impactor (aluminum foil ø25 mm, Dekati, Kangasala, Finland, product code CF-300) were used for the experiment. The substrates were covered with Apiezon-L grease (Dekati, Kangasala, Finland, product code DS-515). The mass of the particles generated during each stage of the impactor was determined by weighing the covered substrates before and after the fire experiment. The filters were weighed using an MYA 5.3Y.F microbalance (1 μg resolution, RADWAG, Radom, Poland). Prior to weighing, the substrates were conditioned for 48 h in a weighing room at a temperature of 20 ± 2 °C and relative air humidity of 50 ± 5%.

The impactor measurement was carried out to determine the NSD and thus the VSD of the particulates emitted during the fire experiments. The substrates attached to the impactor stage were used for the determination of the MSD, which was not recalculated from online impactor measurements but determined by gravimetric measurements. All size distributions were evaluated as cumulative distributions instead of d/dlogDp distributions. Based on this approach, exact values can be maintained while allowing for a better interpretation of diameters, eliminating problems related to the median diameter of the stage, and focusing only on the cutoff diameter. In addition, potential confusion with respect to the assignment of particles on stages can be avoided and the proportions of different populations of particles as well as their densities can be identified.

The analysis of size distributions depends on the choice of the particle diameter definition. In this work, we focused on determining the relative densities of particles emitted during fire experiments. Therefore, all size distributions are discussed as functions of the Stokes diameter [58]. All measured distributions were based on Stokes diameters calculated using the Dekati ELPI+XLS v2.01 software (Dekati, Kangasala, Finland) [58].

### 2.2. Experimental Setup of the Impactor

The NSD and VSD measurements were performed using the HT-ELPI. The HT-ELPI is a 15-stage electrical, low-pressure impactor that is capable of the direct measurement of particles in hot gases without prior cooling, which makes it ideal for the analysis of particles generated during fires. The nominal particle sizes range from 6 nm to 10 µm. The data obtained from the impactor were analyzed using the ELPI+XLS 2.01 data processing spreadsheet [58]. The sampling time of the VSD obtained by using the ELPI is the same as that of the MSD collected on aluminum foils and determined using the microbalance. For each test fire, we obtained 14 data points representing the VSD $\left\{f_{V,i},\;i\in \left[1,14\right]\cap \mathbb{N}\right\}$, expressed in $\frac{{\mu m}^{3}}{{\mathrm{cm}}^{3}}$, using the ELPI+XLS and 14 data points representing the MSD $\left\{f_{m,i},\;i\in \left[1,14\right]\cap \mathbb{N}\right\}$ obtained with the microbalance.

### 2.3. Material Fire Experiments

The fire experiments were conducted in the fire laboratory of The Main School of Fire Service in a chamber designed to evaluate the efficiency of fire detection systems, especially that of smoke detectors. The chamber was approximately 5 m×5 m×3 m in size. Five samples were burned during the experiment.

First, the sample was prepared and weighed, and then placed inside the test chamber. Subsequently, sampling using the HT-ELPI was started. To ignite the sample, 40 mL of denatured alcohol (>97% vol.) was poured onto it and ignited with a gas soldering lamp. After 5 min, the ELPI sampling was stopped and the fire was extinguished with approximately 350 mL of water. The filter stage block was disassembled and greased aluminum foils were replaced with new ones. The chamber was ventilated using a powered ventilation system. While the ELPI sampling was stopped, particle concentration in the chamber was monitored using DustTrak 8534 DRX Aerosol Monitor sampler (TSI, MN, USA), which was calibrated using a previously reported procedure [59]. The ventilation continued until the concentration of particles with diameters less than 10 µm, as measured using DustTrak 8534 DRX (TSI, MN, USA), was less than 10^4^ m^−3^. The procedure was repeated for all five samples.

**Figure 1:**
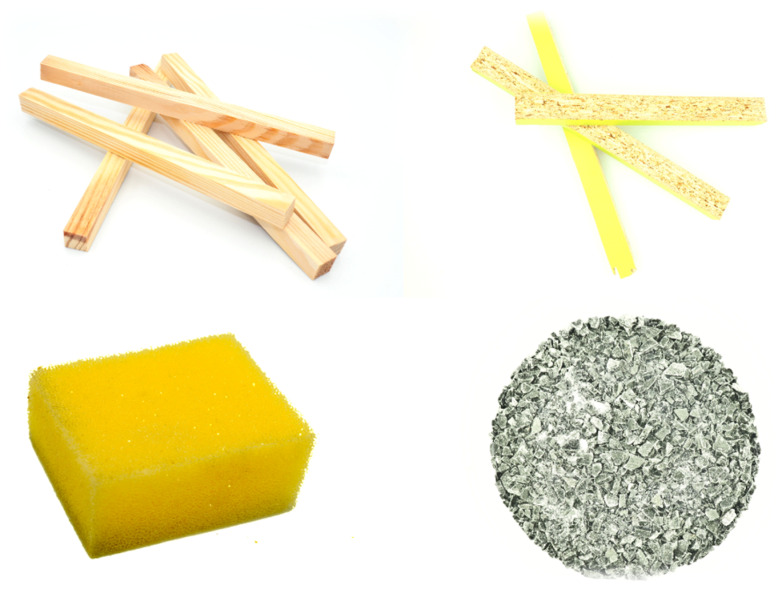
Four materials used in the experiment. The pinewood scantlings (PINE, **upper left**), laminated particleboard (LPB, **upper right**), fragment of PUR foam sheet (**lower left**), granules of PMMA (**lower right**).

The first sample was PINE (50 square cuboids arranged in five layers). The scantlings are presented in Figure 1 (upper left). The scantlings were placed perpendicularly in the neighboring layers. The total mass was 397.7 ± 0.1 g. The second sample was LPB, which was cut into oblong prisms presented in Figure 1 (upper right). The pieces were spaced and layered, allowing for the free flow of air between them. The total mass was 203.7 ± 0.1 g. The third sample was a 50.0 ± 0.1 g PUR foam sheet; a small piece from this sample is presented in Figure 1 (lower left). The PUR foam had a low density and thus the mass of the sample was lower. The sheet was cut into pieces, one piece per layer. The height and perimeter of the sample were similar to those of PINE and LPB; however, there was no airflow between the pieces of the sheet. The fourth sample consisted of PMMA granules with a diameter of <3 mm (Figure 1, lower right), which were arranged in a pile (heap) according to the angle of repose. The total weight was 200.2 ± 0.1 g. The fifth sample consisted of all materials used before, but four times smaller amounts of these materials were used to maintain the order of the number concentration of the particles. The weights of the PINE, LPB, PUR, and PMMA were 102.5 ± 0.1, 49.5 ± 0.1, 12.5 ± 0.1, and 50.1 ± 0.1 g, respectively, leading to a total weight of 214.6 ± 0.4 g. This sample was denoted as MIX. The PUR foam was placed on the bottom of the sample and the PMMA was placed in four low piles. Pieces of the LPB were partially supported on PMMA piles and partially supported on PUR foam; however, they were separated from each other, allowing for airflow. The top of the LPB layer was set roughly horizontally; PINE scantlings were placed on the LPB layer while maintaining the spacing between them.

### 2.4. Size Distribution of Particulates Emitted from Fires

#### 2.4.1. Volume Size Distribution

The data were analyzed using ELPI+VI 2.1 software and ELPI+XLS 2.01 provided by the manufacturer, Dekati (Kangasala, Finland), as well as Python 3 [60]. The ELPI+XLS software was used to read data collected by the HT-ELPI, and it transformed the NSDs into 14-point data sets $\left\{f_{V,i}\right\}$ representing the VSD at each stage of the impactor. The remaining part of the analysis was conducted using Python.

Based on the literature [61,62], the particle sizes can be described using a log-normal distribution; however, in our experiment, we assumed the presence of more than one population of particles. Therefore, our VSD $f_{VSD}\left(x\right)$ is a sum of arbitrary numbers n≥1 of log-normal distributions. The probability density function (PDF) $f_{VSD}\left(x\right)$ (1) and cumulative distribution function $CDF_{VSD}\left(x\right)$ (2) are presented below.

(1) $$f_{VSD}\left(x\right)=\overset{n}{\underset{j=1}{\sum }}f_{VSD}^{j}\left(x\right)=\overset{n}{\underset{j=1}{\sum }}\frac{A_{j}}{\sigma_{j}x}\exp\left(-\frac{{\left(\ln x-\mu_{j}\right)}^{2}}{2\sigma_{j}}\right)$$

(2) $$CDF_{VSD}\left(x\right)=\overset{n}{\underset{j=1}{\sum }}CDF_{VSD}^{j}\left(x\right)=\overset{x}{\underset{0}{\int }}f_{VSD}\left(y\right)dy=\overset{n}{\underset{j=1}{\sum }}\overset{x}{\underset{0}{\int }}\frac{A_{j}}{\sigma_{j}y}\exp\left(-\frac{{\left(\ln y-\mu_{j}\right)}^{2}}{2\sigma_{j}}\right)dy=\overset{n}{\underset{j=1}{\sum }}\frac{a_{j}}{2}\left(1+\mathrm{erf}\left(\frac{\ln x-\mu_{j}}{\sigma_{j}\sqrt{2}}\right)\right),$$

where x is the diameter, A is a proportionality constant, μ is the natural logarithm of the mean diameter, σ is the shape parameter, y is the integration variable, $a_{j}$ is the abundance, and erf is the error function. Each summand in Equation (2) adds three fitting parameters. Hence, the fitting procedure depends on the measured VSD $\left\{f_{V,i}\right\}$ (support of the PDF, number of modes) and measured volume CDF $\left\{CDF_{V,i}=\frac{\sum_{k=1}^{i}f_{V,i}}{\sum_{k=1}^{14}f_{V,i}}\right\}$. When the number of degrees of freedom did not permit the fitting of coefficients and their uncertainties, the fitted function was limited to a smaller number of summands. The $CDF_{VSD}$ was fitted to the measured set $\left\{CDF_{V,i}\right\}$ in Python using the least squares method [63] from lmfit package [64] and erf function from scipy.special [65].

#### 2.4.2. Mass Size Distribution and Density

The MSD of the particles emitted from each test fire was obtained by measuring the increase in the mass of aluminum filters $\left\{f_{m,i}\right\}$ and mass CDF $\left\{CDF_{m,i}=\frac{\sum_{k=1}^{i}f_{m,i}}{\sum_{k=1}^{14}f_{m,i}}\right\}$. The MSD can be derived from the VSD using the density of the substance ρ, as shown in Equation (3). In general, each type of particle (each distribution) has a different density, $\rho_{j}$. The cumulative MSD $CDF_{MSD}\left(x\right)$ can be calculated from the $\rho_{j}$ and cumulative VSD, as shown in Equation (4) ($\rho_{0}$ is a normalization constant).

(3) $$f_{MSD}\left(x\right)=\overset{n}{\underset{j=1}{\sum }}\frac{\rho_{j}}{\rho_{0}}\frac{A_{j}}{\sigma_{j}x}\exp\left(-\frac{{\left(\ln x-\mu_{j}\right)}^{2}}{2\sigma_{j}}\right)=\overset{n}{\underset{j=1}{\sum }}\frac{\rho_{j}}{\rho_{0}}f_{VSD}^{j}\left(x\right)$$

(4) $$\begin{array}{ll} CDF_{MSD}\left(x\right) & =\overset{n}{\underset{j=1}{\sum }}CDF_{MSD}^{j}\left(x\right)=\overset{x}{\underset{0}{\int }}f_{MSD}\left(y\right)dy=\overset{x}{\underset{0}{\int }}\overset{n}{\underset{j=1}{\sum }}\frac{\rho_{j}}{\rho_{0}}f_{VSD}^{j}\left(x\right)dy=\overset{n}{\underset{j=1}{\sum }}\frac{\rho_{j}}{\rho_{0}}\overset{x}{\underset{0}{\int }}f_{VSD}^{j}\left(y\right)dy=\overset{n}{\underset{j=1}{\sum }}\frac{\rho_{j}}{\rho_{0}}CDF_{VSD}^{j}\left(x\right)\\ & =\overset{n}{\underset{j=1}{\sum }}\frac{b_{j}}{2}\left(1+\text{erf}\left(\frac{\ln x-\mu_{j}}{\sigma_{j}\sqrt{2}}\right)\right), \end{array}$$

where $b_{j}$ is the mass abundance. The parameterized MSD $CDF_{MSD}^{j}\left(x\right)$ and parametrized VSD $CDF_{VSD}\left(x\right)$ share shape $\sigma_{j}$ and location parameters $\mu_{j}$. The sum of Equation (4) can be expressed in terms of Equation (2). The $CDF_{MSD}\left(x\right)$ can be fitted to a set of $\left\{CDF_{m,i}\right\}$ using the $b_{j}$ values, as shown in Equation (5). Since $\sum_{j=1}^{n}a_{j}=1$ and $\sum_{j=1}^{n}b_{j}=1$, $b_{l}$ can be expanded, as shown in Equation (6).

(5) $$CDF_{MSD}^{j}\left(x\right)=\frac{\rho_{j}}{\rho_{0}}CDF_{VSD}^{j}\left(x\right)⟹\frac{b_{j}}{2}\left(1+\mathrm{erf}\left(\frac{\ln x-\mu_{j}}{\sigma_{j}\sqrt{2}}\right)\right)=\frac{\rho_{j}}{\rho_{0}}\frac{a_{j}}{2}\left(1+\mathrm{erf}\left(\frac{\ln x-\mu_{j}}{\sigma_{j}\sqrt{2}}\right)\right)⟹b_{j}=\frac{\rho_{j}}{\rho_{0}}a_{j}$$

(6) $$b_{l}=\frac{b_{l}}{\sum_{j=1}^{n}b_{j}}=\frac{\frac{\rho_{l}}{\rho_{0}}a_{l}}{\sum_{j=1}^{n}\frac{\rho_{j}}{\rho_{0}}a_{j}}=\frac{\rho_{l}a_{l}}{\sum_{j=1}^{n}\rho_{j}a_{j}}$$

Equation (5) can be rewritten as a system of equations $\rho_{l}a_{l}=\sum_{j=1}^{n}\rho_{j}a_{j}b_{l}$ in the form of a matrix with unknown densities ρ.

(7) $$\left[\begin{array}{cccc} a_{1}b_{1}-a_{1} & a_{2}b_{1} & \cdots & a_{n}b_{1}\\ a_{1}b_{2} & a_{2}b_{2}-a_{2} & \cdots & a_{n}b_{2}\\ \vdots & \vdots & \ddots & \vdots \\ a_{1}b_{n} & a_{2}b_{n} & \cdots & a_{n}b_{n}-b_{n} \end{array}\right]\left[\begin{array}{c} \rho_{1}\\ \rho_{2}\\ \vdots \\ \rho_{n} \end{array}\right]=0$$

Based on $\sum_{j=1}^{n}a_{j}=1$ and $\sum_{j=1}^{n}b_{j}$, the rank of the left matrix in Equation (7) is equal to n−1; according to the Rouché–Capelli theorem (Kronecker–Capelli theorem), we can express $\left\{\rho_{2},\ldots ,\rho_{n}\right\}$ as a function of $\rho_{1}$.

The results for each family j of particles include four parameters: volume abundance $a_{j}$, mean diameter $D_{m}^{j}=\exp\left(\mu_{j}\right)$, geometric standard deviation $GSD_{j}=\exp\left(\sigma_{j}\right)$, and mass abundance $b_{j}$. If the fitting procedure included n≥2 log-normal distributions, the additional parameter–density $\rho_{j}$, as a function of $\rho_{1}$, was obtained for 2≤j≤n.

#### 2.4.3. Goodness of Fit and Uncertainties

Each fit was evaluated using the reduced chi-square $\chi_{\nu }^{2}=\frac{\chi^{2}}{\nu }$. The fitting procedure minimizes $\chi^{2}$. The number of degrees of freedom ν is the difference between the number of data points (14) and the number of fitted parameters, that is, 3n, based on Equations (2) or (4). For each fit, we provide both $\chi_{\nu }^{2},\;\nu$ and the right-hand side probability p of this value according to the $\chi^{2}$ distribution. In general, very low p values (and $\chi_{\nu }^{2}\gg 1$) represent “underfitted” data, whereas very high p values ($\chi_{\nu }^{2}\ll 1$) represent “overfitted” data. A detailed discussion is provided for each fit.

The fitting procedure in the lmfit package provides uncertainties for the parameters ($a_{j},\;\mu_{j},\sigma_{j}$) fitted during VSD fitting and $b_{j}$ fitted during MSD fitting. The uncertainties of $D_{m}^{j},GSD_{j},\;\mathrm{and}\;\rho_{j}$ must be determined according to [66]. In the case of $D_{m}^{j}$ and $GSD_{j}$, which are one-variable functions of the fitted parameters, the uncertainties can be described by Equations (8) and (9). The uncertainty of the density $\rho_{j}\left(a_{1},\ldots ,a_{n},b_{1},\ldots ,b_{n}\right)$ for j≠1 is provided in Equation (10). Depending on n, the solution of Equation (7) has more or fewer summands in the numerator and denominator and thus Equation (10) becomes more or less complex.

(8) $$\Delta D_{m}^{j}=\left|\frac{\partial D_{m}^{j}}{\partial \mu_{j}}\right|\Delta \mu_{j}$$

(9) $$\Delta GSD_{j}=\left|\frac{\partial GSD_{j}}{\partial \sigma_{j}}\right|\Delta \sigma_{j}$$

(10) $$\Delta \rho_{j}=\sqrt{\overset{n}{\underset{l=1}{\sum }}\left({\left|\frac{\partial \rho_{j}}{\partial a_{l}}\right|}^{2}\Delta a_{l}^{2}+{\left|\frac{\partial \rho_{j}}{\partial b_{l}}\right|}^{2}\Delta b_{l}^{2}\right)}$$

## 3. Results and Discussion

### 3.1. Raw ELPI Result

The first analysis of the data collected by the HT-ELPI was performed using the ELPI+XLS sheet provided by Dekati (Kangasala, Finland). The number concentrations, number median diameters (NMDs), and volume median diameters (VMDs) obtained with this software are presented in Table 1. The total number concentration ranges from 2 to 7 million particles per cubic centimeter. Values of this order (millions of particles per cubic centimeter) were obtained for smoke from cotton smoldering and wood smoke. The NMDs were comparable for all materials (within a factor of two). The highest concentration was obtained for MIX. In this case, the NMD is the highest among the samples, whereas the VMD is lower than that of PMMA. Half of the number of particles emitted from the MIX fire had a diameter less than 115 nm and the total volume was much smaller than half of the total volume of all particles, because half of the volume consists of particles with diameters below 426 nm. The smallest particles represent the largest proportion, whereas their total volume is low; it is likely that their weight is also small. Similar conclusions can be drawn for all other burned materials, that is, the NMD is generally 4–6 times smaller than the VMD. The number concentration, NMD, and VMD of LPB were the smallest among the investigated samples. The NMD ranged from 50 to 100 nm, corresponding to the Aitken nuclei range based on Whitby [67], that is, every second particle emitted during the fire test is a fine particle in the Aitken nuclei range. Furthermore, more than half of the volume of all particles consisted of fine particles because all VMDs are smaller than 2 µm.

### 3.2. Parametrization of the Particulates from Fire Experiments

**Figure 2:**
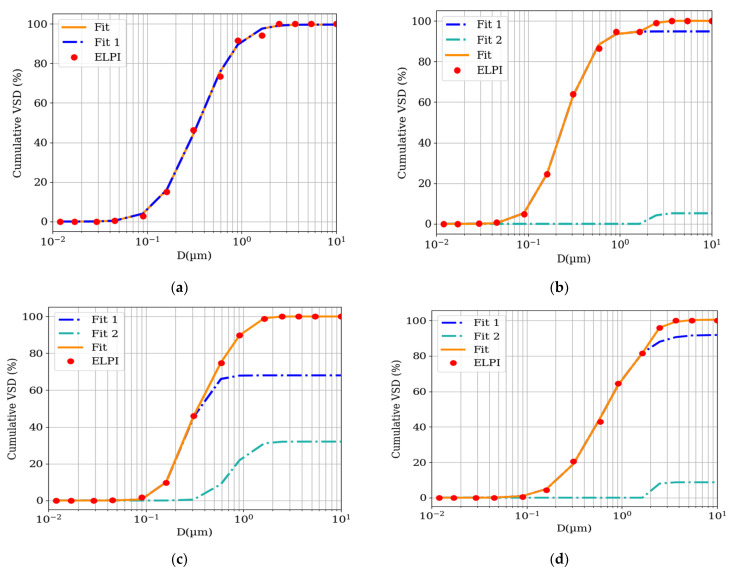
Cumulative volume size distributions of particles emitted during (**a**) pinewood (PINE), (**b**) laminated particle board (LPB), (**c**) PUR foam, and (**d**) PMMA fires. The red dots represent the results obtained with ELPI. The orange line is the best-fit line parametrization of the distribution based on two summands (blue: Fit 1, sea green: Fit 2). In the case of pinewood, it was impossible to fit the sum of two distributions; therefore, the orange line overlaps the blue one.

The cumulative VSD obtained using the HT-ELPI impactor was fitted with the procedure presented in Section 2.4. The fitted sums of the log-normal distributions for PINE (Figure 2a), LPB (Figure 2b), PUR (Figure 2c), and PMMA (Figure 2d) are presented below (denoted as Fit). The fitted sums could be split into separate summands if *n* ≥ 2. Fit 1 in Figure 2 corresponds to the first summand in Equation (2), that is, the summand with index *j* = 1. Fit 2 corresponds to the second summand in Equation (2), with index *j* = 2. The measurements for the PINE sample did not allow the fit of more than an *n* = 1 distribution using Equation (2). In Figure 2b–d, *n* = 2. Hence, the relative density of the particles according to Equation (7) was not determined for PINE, whereas it was used for the other three materials. The fitted numeric abundance (*a*), mean Stokes diameter (*D_m_*) and geometric standard deviation (*GSD*) values are presented in Table 2.

In the case of the PINE sample, the fitted abundance a is within uncertainty equal to 100%, and the relative uncertainty of a is below 1%. The fit statistics are as follows: $\chi_{\nu }^{2}=2.431\;\mathrm{and}\;\nu =14-3=11$. The corresponding p=0.12 is neither low nor high, representing a reliable fit, which can also be confirmed by Figure 2a. The fitted volume abundance value $a_{j}$ also proves that the size distribution can be described well using a single (unimodal) log-normal distribution. These results must be considered together with the resolution of the impactor. If the impactor used in the experiment had more than 15 stages covering a range of 0.01–10 µm, especially in the range 0.1–1 µm, the characteristics of the particles generated during PINE fires can be investigated further. Potentially, the single mode can be split into a few modes that are close to each other if the resolving power of the device (i.e., the stage density) allows it. However, the results show that during the burning of PINE, no particles with diameters in the range of 2.5 to 10 µm were emitted, and more than 90% of the volume of all measured particles was between 100 nm and 1 µm.

The results from the studies in which the Dekati low-pressure impactor (DLPI) was used to determine the MSD of particles emitted during the pyrolysis of PINE in a furnace at 1300 °C [41] showed that the majority of the mass consisted of particles with diameters between 20 nm and 500 nm. The difference between the results obtained in the present study and the previous work [41] shows that two modes exist. That is, the particles were in the nucleation mode in the furnace studies [67], whereas with respect to open burning, as could be expected, the burning was incomplete, the emitted particles were not products of complete thermal decomposition, and the products were mainly in the accumulation mode [67].

In the case of the PINE sample, it was impossible to evaluate the size distribution as the sum of more than one log-normal distribution, whereas the data for all remaining samples were analyzed as the sum of two log-normal distributions.

Figure 2b presents the VSD for the fire experiment using the LPB sample. The fit of the sum of two log-normal distributions to the data from the ELPI yielded the following values: $\chi_{\nu }^{2}=0.6847,\;\nu =14-6=8$, and p=0.9996. As indicated in Section 2.4.3, this *p*-value is high. Although one can use the one log-normal distribution for the fit, which has a value of $\chi_{\nu }^{2}$ closer to 1 than that presented in the fit in Figure 2b, the increase in the diameters (1.6 and 2.5 µm) shows that fitting Equation (2) with n=1 is not suitable. The fit parameters are presented in Table 2. The log-normal distributions, Fits 1 and 2, have abundances of 94.8 ± 0.6% and 5.2 ± 0.8%, respectively, leading to a sum of 100.0 ± 1.4%. Although the sum is correct, with a small uncertainty, there are problems with respect to the shape and location parameters of Fit 2. Based on the resolution of the impactor, $D_{m}^{2}\in \left[1.6,3.6\right]\;\mu m$, that is, between Stokes cut-off diameters of stage 11 and stage 13 of the impactor. Therefore, we cannot provide the $D_{m}^{2}$ and its uncertainty as well as $GSD_{2}$ and its uncertainty. The value of 2.48 in Table 2 is only included for location purposes because it was used in the equation for Fit 2 and thus for the best-fit line in Figure 2b. In the case of LPB, around 95% of the volume of particles is from particles with a diameter less than 1 µm.

However, uncertainties in $D_{m}$ and GSD are not required to determine the relative densities based on Equation (7) and their uncertainties based on Equation (10). The mass abundances $b_{j}$ are obtained by fitting the cumulative MSD using Equation (4) with the $\mu_{j}$ and $\sigma_{j}$ values obtained from the fit of the cumulative VSD using Equation (2), that is, during the fit of the cumulative MSD, only the $b_{j}$ values are obtained. The results of the LPB fit are presented in Table 2. Although Fit 2 constitutes a small proportion of the volume of the particulates (5.2 ± 0.8%), its proportion in the mass of the particles is approximately five times higher. Hence, the calculated density of the particles described by Fit 2 is $\rho_{2}=\left(6.74\pm 1.04\right)\cdot \rho_{1}$. The $\rho_{2}$ value is high because if we assume that $\rho_{1}=1\frac{g}{{\mathrm{cm}}^{3}}$, $\rho_{2}=6.74\pm 1.04\frac{g}{{\mathrm{cm}}^{3}}$. This value can be explained by the high abundance of metal particles. This is questionable because there are few substances, such as metals, that have a density of this order. The generation of pure metal particles in LPB fires can rather be excluded. Further experiments covering the region between 1.6 and 3.6 µm could reveal the true shape and location of Fit 2; without this the $\rho_{2}$ value cannot be determined more accurately.

The third sample burned in our experiment was PUR foam. The visible discrepancies between the data and fitted one log-normal distribution suggest that the VSD is a result of the sum of more distributions. The $\left\{f_{V,i}\right\}$ values measured using the ELPI allow for the fit of the CDF with n=2 (Figure 2c); however, uncertainties were determined for all fit parameters. The volume abundances of Fits 1 and 2 have a 2:1 ratio (Table 2) and significantly higher uncertainties; however, the sum equals 100 ± 26%. The reduced chi-square of the fit is $\chi_{\nu }^{2}=0.2364,\;\nu =14-6=8,\mathrm{and}\;p=0.999993$, representing an overpredictive model. However, these values were accepted as the best-fit data because the data fit well and the uncertainties of all parameters are determined. The fit of the cumulative MSD shows that the mass proportions of Fits 1 and 2 are very similar to the volume proportions. However, the sum equals 98.9 ± 19.5%, which is correct within uncertainty. Because the mass proportions are equal to the abundances of Fits 1 and 2, the densities of both types of particles are, within uncertainty, equal, that is, $\rho_{2}=\left(0.91\pm 0.42\right)\cdot \rho_{1}$. The high relative uncertainty of $\rho_{2}$ (46%) is due to the high relative uncertainties of the fitted volume and mass abundances $a_{j}$ and $b_{j}$, which exceed 30% of the fitted value for Fit 2. This might be due to the resolution of the impactor. An increase in the number of stages will lead to intermediate points and thus a higher fit quality. The majority of particles emitted from burning PUR are in the range of 100 nm to 2 µm, and the proportion of particles measured by the impactor to be bigger than 2 µm or smaller than 100 nm is insignificant.

The PUR is widely used for padding upholstered furniture; therefore, the products of its thermal decomposition have been widely investigated. This was discussed in the context of room fire experiments and smoke detector performance [68]. The particles emitted from these experiments were analyzed using the ELPI (non-HT version). It is difficult to compare this experiment to ours because the methodology used for the mass determination was poorly described and no distributions were presented therein.

The fourth investigated material was PMMA. The cumulative VSD is significantly shifted toward higher diameters compared with all other samples. The data collected by the ELPI permitted fitting the sum of two log-normal distributions, Equation (2). In this case, $\chi_{\nu }^{2}$ is almost equal to 1, representing a perfect fit ($\chi_{\nu }^{2}=0.9866,\;\nu =14-6=8,\;p=0.998$, Figure 2d). Unfortunately, similar to the LPB sample, the location and shape parameters of Fit 2 could not be precisely determined, and thus the diameter $D_{m}$ and GSD were roughly estimated (Table 2). In this case, $D_{m}$ ranges between stages 11 and 13 of the impactor, that is, between 1.6 and 3.6 µm, respectively. In contrast to previous sums of distributions, the sum of the volume abundances exceeds 100%, that is, 100.4 ± 5.1%, which is still correct within uncertainty. The fit of the cumulative MSD resulted in values of mass abundances $b_{j}$ which summed within uncertainty to 100% (i.e., 98.3 ± 3.7%). Similarly, as in the case of PUR, the mass abundances $b_{j}$ are similar to the volume abundances $a_{j}$. The particle densities obtained by Fits 1 and Fit 2 can be assumed to be equal within the uncertainty $\rho_{2}=\left(1.12\pm 0.33\right)\cdot \rho_{1}$.

The MSD of the particles emitted from burning PMMA was previously discussed [69]. The distributions presented in this study show that two types of particles are emitted from the PMMA fire: (1) particles with a mean diameter of 0.1–0.3 µm; and (2) particles with a mean diameter of 1–3 µm. These values were obtained for ventilated combustion using a cone calorimeter. The location of the second type of particles is in agreement with our study; the coarse particles [67] emitted during the burning of PMMA are similar regardless of the burning condition. The location of the first type of particles differs in our experiment, which might be due to partial thermal decomposition and the observation of accumulation particles [67], whereas particles in [69] shifted toward nucleation particles [67].

#### Predicting VSD of Particles

**Figure 3:**
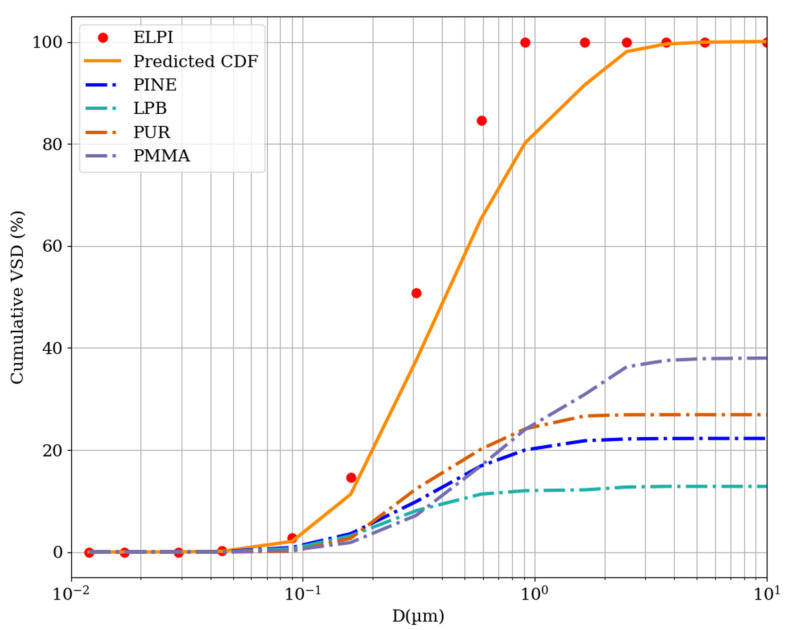
Cumulative volume size distribution of particles emitted during the MIX fire. The red dots represent values measured with the ELPI. The orange line is the predicted CDF line, which is the normalized sum of the four CDFs of the raw materials (dash-dotted lines; blue—pinewood, sea green—particle board, dark orange—PUR foam, and medium purple—PMMA).

To evaluate the results of the fire experiments on four raw material samples, we prepared and burned a sample containing all raw materials (MIX). The masses of the ingredients were four times smaller than those used for the burning of pure materials. The cumulative VSD of the particles emitted during the burning of MIX should be a weighted sum of the parametrized distributions for the separate materials. As the weight, we used the concentration (C) of the given material divided by the sum of the concentrations of all materials (presented in Table 1), as described in Equation (11). This normalization of the CDF of MIX should result in a good CDF estimate based on the CDFs of the pure materials. The cumulative VSD obtained from ELPI measurements is presented in Figure 3, together with the volume CDF estimated from Equation (11), which can be decomposed into separate summands corresponding to the pure materials.

(11) $$CDF_{VSD}^{MIX}\left(x\right)=\frac{C^{PINE}\cdot CDF_{VSD}^{PINE}\left(x\right)+C^{PB}\cdot CDF_{VSD}^{PB}\left(x\right)+C^{PUR}\cdot CDF_{VSD}^{PUR}\left(x\right)+C^{PMMA}\cdot CDF_{VSD}^{PMMA}\left(x\right)}{C^{PINE}+C^{PB}+C^{PUR}+C^{PMMA}}$$

There is a systematic discrepancy between the predicted and measured ELPI values. The CDF of MIX is shifted toward smaller diameters compared with the predicted CDF, which might have many reasons. One possible reason for this is the decomposition temperature. Based on the differential scanning calorimetry results, the thermal decomposition of PINE starts at 150 °C [37], whereas LPB burns at temperatures higher than raw wood [42], and PUR foam and PMMA are burned at temperatures higher than 300 °C [70,71]. Therefore, the thermal decomposition of PINE in MIX will occur at a higher temperature than in the PINE fire, the decomposition will be less partial, and the distribution of particles emitted in such a fire will be shifted to smaller diameters. We cannot evaluate the exact shifts of the CDFs toward lower diameters; however, the measured and predicted CDFs fall into the Aitken particle range [67]. The difference observed for higher diameters and the lack of coarse particles (diameter higher than 1 µm) show that particles emitted from the MIX fire are the result of a more complete decomposition of materials.

## 4. Conclusions

The results of our experiments depend on our specific experimental setup. Although our chamber was relatively small, spatial nonuniformities might have affected the derived number concentrations. In addition, the arrangement of the sample before burning might affect the way it burns and thus the particle emission. Nevertheless, the research allowed us to draw following conclusions:

1. The mathematical limitations do not allow for the determination of the absolute density of particles based on comparing VSD and MSD. However, it has been proven that the densities of the particles can be expressed as a function of the density of the first type of particles if at least two types of particles can be distinguished (i.e., the fit contains at least two summands). The use of VSD and MSD in this work shows that it is possible to provide more precise results by violating the approach in which a constant density is assumed for all particulates.
2. The relative density obtained for one population of particles, from the fire of LPB, is questionable, which is mainly due to the resolving power of the distribution measured by the impactor.
3. The results show that the use of the cascade impactor with only 15 stages is adequate, even for the determination of six parameter distributions; however, it should be treated as the edge of applicability if more than 90% of the volume of particles is in the range of 100 nm to 1 µm.
4. The prediction of the VSD from the burning of the mixture of materials based on the VSD of the raw material led to a distribution shift toward larger Stokes diameters than that measured with the impactor, which indicates a more complete thermal decomposition during the MIX fire because LPB decomposes at higher temperatures than raw wood. Another possible cause is the interference between the decomposition products of different materials.

The results of our research open two further directions of study. Firstly, the aerosols from construction waste fires can be investigated in the range of 0.01–10 µm, providing more precise values of the parameters determined in this study; secondly, the cross-interactions of products emitted from different materials can be investigated using differential scanning calorimetry (DSC) and thermogravimetry (TG) analyses.

## Figures and Tables

**Table 1:** The number concentration of particles (*C*), number median diameter (NMD), and volume median diameter (VMD) obtained using HT-ELPI for pinewood (PINE), laminated particle board (LPB), polyurethane (PUR), poly(methyl methacrylate) (PMMA), and the sample containing all materials (MIX).

Parameter	Pinewood	LPB	PUR	PMMA	MIX
*C* (cm^−3^)	3.53 × 10^6^	2.03 × 10^6^	4.25 × 10^6^	5.97 × 10^6^	6.54 × 10^6^
NMD (µm)	0.058	0.045	0.077	0.109	0.115
VMD (µm)	0.359	0.277	0.365	0.666	0.426

**Table 2:** Fitted volume abundance *a*, mean diameter *D_m_*, geometric standard deviation *GSD*, and mass abundance *b* of the particles emitted during pinewood (PINE), laminated particle board (LPB), polyurethane (PUR), and poly(methyl methacrylate) (PMMA) fires.

Material	Parameter	Fit 1	Fit 2
PINE	*a* (%)	99.6 ± 0.7	−
	*D_m_* (µm)	0.344 ± 0.008	−
	*GSD*	2.15 ± 0.06	−
LPB	*a* (%)	94.8 ± 0.6	5.2 ± 0.8
	*D_m_* (µm)	0.238 ± 0.003	2.38 ^1^
	*GSD*	1.81 ± 0.03	1.05 ^1^
	*b* (%)	73.6 ± 3.4	27.0 ± 4.0
PUR	*a* (%)	68 ± 13	32 ± 13
	*D_m_* (µm)	0.26 ± 0.03	0.75 ± 0.15
	*GSD*	1.55 ± 0.08	1.5 ± 0.2
	*b* (%)	68.9 ± 9.5	30 ± 10
PMMA	*a* (%)	91.8 ± 2.6	8.6 ± 2.5
	*D_m_* (µm)	0.60 ± 0.03	2.45 ^1^
	*GSD*	2.27 ± 0.09	1.05 ^1^
	*b* (%)	88.8 ± 1.6	9.5 ± 2.1

^1^ This value is for location purposes only and should be treated as approximate. It is caused by the characteristics of the impactor.

## Data Availability

The data presented in this study are available on request from the corresponding author.
